# Stem Cell Based Drug Delivery for Protection of Auditory Neurons in a Guinea Pig Model of Cochlear Implantation

**DOI:** 10.3389/fncel.2019.00177

**Published:** 2019-05-14

**Authors:** Verena Scheper, Andrea Hoffmann, Michael M. Gepp, André Schulz, Anika Hamm, Christoph Pannier, Peter Hubka, Thomas Lenarz, Jana Schwieger

**Affiliations:** ^1^Department of Otolaryngology, Hannover Medical School, Hanover, Germany; ^2^Cluster of Excellence ‘Hearing4all’, German Research Foundation, Bonn, Germany; ^3^Lower Saxony Centre for Biomedical Engineering, Implant Research and Development (NIFE), Hanover, Germany; ^4^Department of Orthopaedic Surgery, Hannover Medical School, Hanover, Germany; ^5^Fraunhofer Institute for Biomedical Engineering IBMT, Sulzbach, Germany; ^6^Fraunhofer Project Center for Stem Cell Process Engineering, Würzburg, Germany; ^7^Department of Experimental Otology, Hannover Medical School, Hanover, Germany

**Keywords:** spiral ganglion neuron, functionalized cochlear implant, biological functionalization, hydrogel, encapsulation, coating, injection, genetically modified cells

## Abstract

**Background:** The success of a cochlear implant (CI), which is the standard therapy for patients suffering from severe to profound sensorineural hearing loss, depends on the number and excitability of spiral ganglion neurons (SGNs). Brain-derived neurotrophic factor (BDNF) has a protective effect on SGNs but should be applied chronically to guarantee their lifelong survival. Long-term administration of BDNF could be achieved using genetically modified mesenchymal stem cells (MSCs), but these cells should be protected – by ultra-high viscous (UHV-) alginate (‘alginate-MSCs’) – from the recipient immune system and from uncontrolled migration.

**Methods:** Brain-derived neurotrophic factor-producing MSCs were encapsulated in UHV-alginate. Four experimental groups were investigated using guinea pigs as an animal model. Three of them were systemically deafened and (unilaterally) received one of the following: (I) a CI; (II) an alginate-MSC-coated CI; (III) an injection of alginate-embedded MSCs into the scala tympani followed by CI insertion and alginate polymerization. Group IV was normal hearing, with CI insertion in both ears and a unilateral injection of alginate-MSCs. Using acoustically evoked auditory brainstem response measurements, hearing thresholds were determined before implantation and before sacrificing the animals. Electrode impedance was measured weekly. Four weeks after implantation, the animals were sacrificed and the SGN density and degree of fibrosis were evaluated.

**Results:** The MSCs survived being implanted for 4 weeks *in vivo*. Neither the alginate-MSC injection nor the coating affected electrode impedance or fibrosis. CI insertion with and without previous alginate injection in normal-hearing animals resulted in increased hearing thresholds within the high-frequency range. Low-frequency hearing loss was additionally observed in the alginate-injected and implanted cochleae, but not in those treated only with a CI. In deafened animals, the alginate-MSC coating of the CI significantly prevented SGN from degeneration, but the injection of alginate-MSCs did not.

**Conclusion:** Brain-derived neurotrophic factor-producing MSCs encapsulated in UHV-alginate prevent SGNs from degeneration in the form of coating on the CI surface, but not in the form of an injection. No increase in fibrosis or impedance was detected. Further research and development aimed at verifying long-term mechanical and biological properties of coated electrodes *in vitro* and *in vivo*, in combination with chronic electrical stimulation, is needed before the current concept can be tested in clinical trials.

## Introduction

The cochlear implant (CI) is the standard treatment for unilateral and bilateral severe to profound sensorineural hearing loss, both in adults and children. More than 350,000 deaf individuals have already received cochlear implantations worldwide (NIDCD, 2017). In this device, acoustic signals are detected by a microphone, converted into electrical signals and transmitted transcutaneously to an implanted receiver. The signal is decoded and delivered via an electrode array implanted into the scala tympani of the cochlea to the auditory nerve. The entire frequency range of the acoustic signal is split into different frequency bands and allocated to the different contacts, mimicking the physiological tonotopic organization of the cochlea (Lenarz and Scheper, 2015).

The loss of hair cells associated with deafness is followed by retraction of the peripheral nerve fibers in both the animal model (Zilberstein et al., 2012) and in humans (Liu et al., 2015; Whitlon, 2017), and then by degeneration of spiral ganglion neuron (SGN) cell bodies (Liu et al., 2015). This secondary degeneration is highly dependent on the cause of hearing loss and the cochlear structures affected. However, the number of SGNs is crucial for the success of cochlear implantation (Seyyedi et al., 2014). Thus, the prevention of progressive SGN degeneration is a major goal of CI research. The electrical stimulation of SGNs by the CI may (*per se*) reduce the degeneration of SGNs via depolarization-induced neurotrophic signaling pathways (Hansen et al., 2001; Scheper et al., 2009; Leake et al., 2013). The extent to which electrical stimulation alone is able to protect SGNs from degeneration *in vivo* (Li et al., 1999; Agterberg et al., 2010) is unknown, as is the dependence of the protective mechanism of electrical stimulation on various factors such as the onset and duration or stimulation parameters used (Araki et al., 1998; Leake et al., 1999).

In addition to electrical stimulation via the CI, neuroprotective effects on SGNs have also been demonstrated for the application of exogenous neurotrophic factors both *in vitro* (Hegarty et al., 1997) and *in vivo* (Scheper et al., 2009; Leake et al., 2011). The neuroprotective effect of neurotrophic factors persists a few weeks after cessation of neurotrophic treatment (Maruyama et al., 2008; Agterberg et al., 2009). However, neurotrophic therapies may require ongoing administration if a lifelong survival effect is to be achieved in human patients (Gillespie et al., 2003; Gillespie and Shepherd, 2005). Various systems delivering neurotrophins and other drugs locally to the inner ear are under investigation (El Kechai et al., 2015; Nguyen et al., 2017; Mäder et al., 2018; Hao and Li, 2019). For human use, however, most approaches – if intended for continuous application – are not practicable due to the fact that the application has to be repeated [single injection via needle or catheter-based (Prenzler et al., 2018)], or the device or matrix has to be refilled periodically [osmotic pumps (Brown et al., 1993), or intratympanic hydrogels applied to the round window (Wang et al., 2011)].

Cell-based drug delivery is an alternative approach to chronically treating inner ear neurons. Inoculation of the inner ear with appropriate viral vectors, in order to transduce cochlea cells to over-express a desired neurotrophic factor (Geschwind et al., 1996; Kanzaki et al., 2002), allows long-term stable application without a permanent opening of the cochlea. However, there are several safety concerns (David and Doherty, 2017), such as control of neurotrophic factor dosage, choice of transfection site/volume and, where appropriate, options for preventing expression of factors after transduction (Sacheli et al., 2013).

Another approach to supplying inner ear neurons with cell-based neurotrophic factor is the implantation of autologous, allogenic, or xenogenic cells. Implanted cells can either be genetically engineered to overexpress a desired protein or (*per se*) to produce factors at a neuroprotective concentration. Fibroblasts induced to produce brain-derived neurotrophic factor (BDNF), a neurotrophin, have been shown to protect SGNs from degeneration in guinea pigs (Warnecke et al., 2012; Gillespie et al., 2015). However, implanted cells need to be entrapped into a matrix to avoid uncontrolled migration and to shield them from the host’s immune system (Warnecke et al., 2012). Gillespie et al. (2015) solved this problem by encapsulating fibroblasts into a non-biodegradable, biocompatible alginate matrix (Immupel^TM^, Living Cell Technologies Limited). The same hydrogel was used to encapsulate Schwann cells genetically modified to overexpress the neurotrophins BDNF or neurotrophin 3 (NT-3). These entrapped cells supported SGN survival in an *in vitro* model of deafness (Pettingill et al., 2008) and, in the case of the BDNF-producing Schwann cells, protected SGNs in a guinea pig model (Pettingill et al., 2011).

Choroid plexus cells that natively produce neuroprotective factors to protect inner ear neurons were implanted in deafened cats using unspecified alginate capsules (Skinner et al., 2009; Wise et al., 2011). The encapsulated choroid plexus cells were harvested from pigs and produced a cocktail of various neurotrophic factors including GDNF, BDNF, and VEGF (Skinner et al., 2009). These cells did not protect neurons from degeneration in the animal model used. Combined with electrical stimulation by the CI, however, capsule implantation resulted in improved neuronal survival (Wise et al., 2011). In general, microspheres have the disadvantage that they cannot be easily explanted and replaced, which – considering the timescale of lifelong implantation – is likely to be necessary in human CI users. This constraint can be overcome with cells encapsulated in explantable matrixes such as hollow-fiber membrane capsules equipped with a tether for removal. While these devices have already been successfully used in deafened guinea pigs for SGN protection (Fransson et al., 2018), they induced an increase in foreign-body reaction in deafened cats (Konerding et al., 2017). An alternative approach to the implanting of cells for chronic drug delivery to the inner ear neurons involves adhesion of the cells onto the CI surface. Using ultra-high viscous alginate (UHV-alginate) made of the brown algal species *Lessonia nigrescens* and *Lessonia trabeculata*, it has been shown that coating the CI with this alginate is possible (Schwieger et al., 2018). It has also been demonstrated that BDNF-overexpressing murine fibroblasts survive in the UHV-alginate and release BDNF at a concentration that is neuroprotective *in vitro* (Hütten et al., 2013). Since gaining approval for use in humans of murine fibroblasts as xenogeneic cells may be difficult, a human cell source may prove more beneficial. Human mesenchymal stem cells (MSCs) are a promising alternative for lifelong factor delivery. Genetically modified MSCs overexpressing BDNF are shown to produce BDNF at a neuroprotective concentration *in vitro* (Schwieger et al., 2018). When these cells, too, are encapsulated in UHV-alginate, they rescue SGNs from degeneration *in vitro*. Here we investigate the potential neuroprotective effect of MSCs incorporated into a UHV-alginate matrix in deafened guinea pigs. CI electrodes are coated with the cell-UHV-alginate hydrogel layers and implanted into the scala tympani. Additionally, the UHV-alginate-MSC matrix was injected into the scala tympani and gelled instead of being used to coat the CI. Injection into the inner ear was an approach used not only in deafened animals, but also in hearing animals, to investigate the effect of injection on hearing ability.

## Materials and Methods

### Animals and Experimental Conditions

Adult male t Dunkin-Hartley guinea pigs (*N* = 43, weight 300–500 g, Charles River Laboratories, Sulzfeld, Germany) were kept in a temperature- and humidity-controlled room, exposed to a 24-h light-dark cycle (14 h/10 h) with free access to food and water.

**FIGURE 1:**
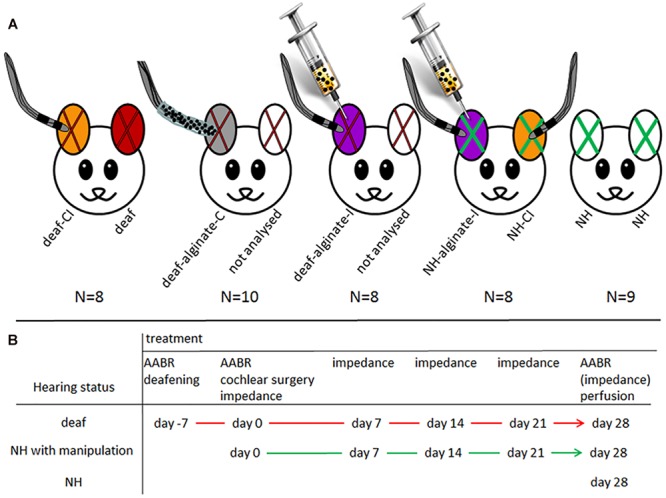
**(A)** Illustration of experimental groups: red X: deafened ears; green X: normal-hearing ears; color code for ears: orange: deafened (red X) or normal-hearing (green X) ears with cochlear implant: *deaf-CI and NH-CI*; red: deafened ear without further treatment, included in group *deaf*; gray: deaf and implantation of CI with UHV-alginate-MSC coating: *deaf-alginate-C*; white with red x: contralateral ears of those treated with factor-releasing cells. Since it cannot be ruled out that the factor has an effect on contralateral neurons, theses ears were not included in the analysis. Violet: animals first received an alginate-MSC injection using a microcatheter system (provided by MED-EL Corp.) inserted 3 mm deep into the scala tympani. After injection the catheter was removed, a normal CI was inserted and the polymerization solution for alginate crosslinking was applied for 30 min at the round window niche. Violet with red X: *deaf-alginate-I*; violet with green X: normal hearing with CI and UHV-alginate-MSC injection: *NH-alginate-I*; white with green X: normal hearing: *NH*. **(B)** illustrates the time line of the experiments for each treatment condition.

All animals were normal hearing, this having been proven by initial measurement of the acoustically evoked auditory brainstem response (AABR, see below). The guinea pigs were randomly divided into five experimental groups. Twenty-six animals were systemically deafened (see below). Deafening was verified after 1 week by AABR measurements, and these animals were randomly assigned to one of three experimental groups unilaterally implanted with the following: a cochlear implant (CI) (*N* = 8; one ear deaf: deaf; one ear deaf and CI inserted: deaf-CI), a CI with alginate-mesenchymal stem cell (MSC) coating (*N* = 10; one ear deaf: not analyzed; one ear deaf with alginate-MSC-coated CI: deaf-alginate-C) or an alginate-MSC injection into the scala tympani followed by the CI insertion (*N* = 8; one ear deaf: not analyzed; one ear deaf with alginate injection*: deaf-alginate-I*). The 17 remaining, non-deafened, normal-hearing animals were either directly sacrificed after verification of normal hearing (*NH; N* = 9) or received a bilateral CI implantation with an additional unilateral alginate-MSC injection (*N* = 8; one ear normal hearing with CI: *NH-CI*; one ear normal hearing with CI and MSC-UHV-alginate injection: *NH-alginate-I*). Figure 1 illustrates the experimental conditions (A) and the time line (B).

The purpose of the various experimental conditions is to provide information on:

(1) Spiral ganglion neuron (SGN) density in normal-hearing ears: NH.
(2) Influence of CI insertion on SGN density and hearing status in normal-hearing ears: *NH-CI*.
(3) SGN density after deafening: *deaf*.
(4) Effect of CI insertion on SGN density in deafened animals: *deaf-CI*.
(5) Neuroprotective potential of CI coated with UHV-alginate containing brain-derived neurotrophic factor- (BDNF-)overexpressing MSCs: *deaf-alginate-C* versus *deaf-CI*.
(6) Neuroprotective potential of UHV-alginate containing BDNF-overexpressing MSCs injected into the inner ear with subsequent CI insertion: *deaf-alginate-I* versus *deaf-CI*.
(7) Influence on hearing threshold of UHV-alginate injection with subsequent CI insertion: *NH-alginate-I*.
(8) Assessment of which application method (coating versus injection) is more favorable: *deaf-alginate-C* versus *deaf-alginate-I*.

Deafening, AABR measurement, inner ear surgery and perfusion were performed under general anesthesia with medetomidine hydrochloride (0.2 mg/kg, intramuscular; CP-Pharma Handelsgesellschaft, Burgdorf, Germany), midazolam (1 mg/kg, intramuscular; Ratiopharm, Ulm, Germany) and fentanyl (0.025 mg/kg, intramuscular; Janssen-Cilag, Neuss, Germany). Animals were placed on a heating pad to maintain the body temperature at 37–38°C. They subcutaneously received 0.05 mg/kg atropine (B. Braun, Melsungen, Germany) to reduce bronchial secretion and salivation, 0.2 mg meloxicam/kg (Boehringer Ingelheim, Ingelheim am Rhein, Germany) for analgesia, and 2 × 4 ml Ringer’s solution including 5% glucose (both from B. Braun) per 300 g body weight, 10 mg enrofloxacin/kg (Bayer Vital, Leverkusen, Germany) for prophylactic antibiotic therapy. Areas to be incised were locally infiltrated with prilocaine (Xylonest 1%, AstraZeneca).

The anesthesia was antagonized by injecting atipamezole (1 mg/kg; Zoetis, Parsippany, United States), flumazenil (0.1 mg/kg; Hexal, Holzkirchen, Germany) and naloxone (0.03 mg/kg, Ratiopharm).

### AABR Measurement

Acoustic stimulation and recording of the auditory brainstem response (AABR) signals were performed using an Audiology Lab system (Otoconsult, Frankfurt a. M., Germany) in a soundproof booth. To detect general auditory system thresholds, acoustic clicks (duration: 50 μs) were used. For detection of frequency-specific acoustic thresholds, tone bursts (duration: 6 ms with 2 ms rising/falling ramps) at frequencies of 1, 2, 4, 8, 16, and 32 kHz with 1 octave step were used. The acoustic stimuli were presented by a calibrated loudspeaker (DT48, BeyerDynamic, Heilbronn, Germany) via a plastic cone placed in the outer ear canal.

The AABR signals were recorded using subcutaneous electrodes. The signals were amplified, band-pass filtered and recorded at a sampling rate of 100 kHz. The signals were analyzed using custom-made software in MATLAB (Mathworks, Natick, MA, United States). The signals were averaged and smoothed using the Savitzky–Golay FIR filter (frame length: 1 ms; polynomial order: 5). The hearing thresholds were determined by visual inspection of AABR signals. The lowest stimulus intensity at which AABR signals could be detected was taken to be a hearing threshold for the relevant stimulus configuration.

Only animals with initial normal hearing (thresholds of <40 dB SPL) were included into the study.

Additional AABR measurements were performed in all animals 1 week after the deafening procedure on experimental day 0 to verify deafness, and in all animals on experimental day 28. In normal-hearing animals, frequency-specific stimulation was performed on day 0 and day 28 (following click measurement) to identify the frequency-specific impact of cochlear manipulation.

The threshold shift was calculated as the difference between the initial hearing threshold and the hearing threshold after deafening or after cochlear implantation in the normal-hearing animals. Where the AABR threshold could not be identified up to the maximum click level [0 dB att. (=120 dB SPL)], the threshold shift was defined as the difference between the AABR threshold at initial measurement and the maximum click level.

### Deafening

Directly after verification of normal hearing by AABR measurement, 26 animals were systemically deafened by subcutaneous injection of kanamycin (400 mg/kg; Kanamycin Sulfate, BioChemica, AppliChem GmbH, Darmstadt, Germany) and subsequent infusion of furosemide (100 mg/kg; Diuren, WDT, Garbsen, Germany) into the external jugular vein (Meyer et al., 2012), which has been shown to eliminate the majority of both inner and outer hair cells (Versnel et al., 2007). The success of the procedure was determined after 1 week by click-evoked AABR measurement. A click AABR threshold shift of 50 dB after the ototoxic treatment was set as the limit for indication of a successful deafening (Meyer et al., 2012). A click AABR threshold shift of 50 dB after the ototoxic treatment was set as the limit for indication of successful deafening (Meyer et al., 2012).

### Preparation of Genetically Modified MSCs

The expression of human BDNF (entire coding sequence including signal peptide: Warnecke et al., 2012) was under the control of a spleen focus-forming virus (SFFV) promoter in a lentiviral vector that also mediated red fluorescence using the marker protein tdTomato (red). Subsequently, after lentivirus production, hMSCs from one selected donor were seeded at 3,000 cells/cm^2^, passage 4 or 5, and were infected with the BDNF-lentivirus including 8 μg/ml polybrene. In order to subsequently downgrade the cells to S1 level, the cells were cultured and expanded for 11 days before being harvested with trypsin/EDTA solution. The medium used for expansion of MSCs (‘MSC medium’) was Dulbecco’s Modified Eagle’s Medium (1 g/l glucose, Biochrom, FG0415) supplemented with 10% (v/v) fetal calf serum (FCS, not heat-inactivated, Thermo Fisher Scientific, Schwerte, Germany, ‘HyClone’, SV30160.03), 25 mM HEPES (Biochrom, Berlin, Germany), 1% (100 U/ml/100 μg/ml) penicillin/streptomycin (Biochrom, Berlin, Germany) and 2 ng/ml human recombinant FGF 2 (from *Escherichia coli*, PeproTech, Hamburg, Germany).

### Preparation of UHV-Alginate-MSC Injections and CI Coating

For injection into the inner ear, 1 ml UHV-alginate solution (0.65% (w/v%) in isotonic 0.9% sodium chloride solution (B. Braun), provided by Fraunhofer IBMT, Sulzbach, Germany, now commercially available from Alginatec GmbH, Riedenheim, Germany) was mixed with 250,000 BDNF-producing MSCs. The alginate-MSC solution was freshly prepared during surgery; immediately following its preparation, it was injected into the scala tympani using a catheter system provided by MED-EL, Innsbruck, Austria. Two catheter types were used, one with a yellow conus and an outer diameter of 0.38 mm, and one with a transparent conus and an outer diameter of 0.64 mm. The thinner catheter had a more flexible consistency and was more difficult to insert, but was still the first choice as its insertion is hypothetically less traumatizing than that of the transparent catheter system.

**FIGURE 2:**
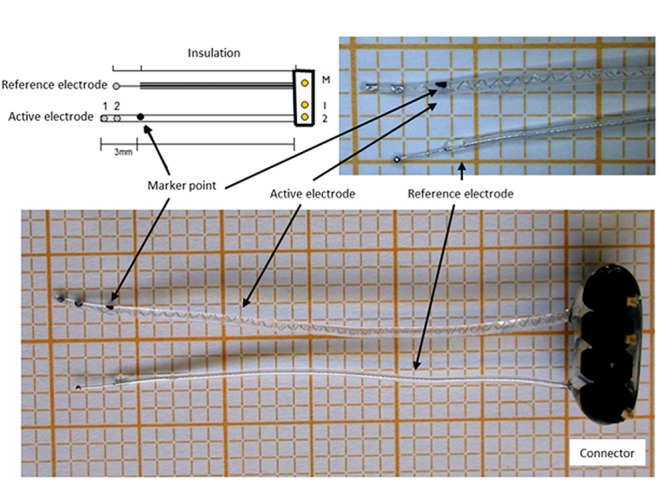
Cochlear implant electrode. The electrodes consisted of a connector, a reference electrode and an active electrode with two contacts and a marker point to determine the insertion depth.

**FIGURE 3:**
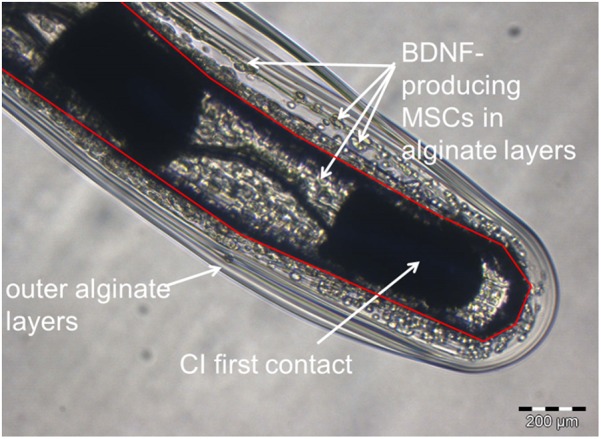
Representative image of a UHV-alginate-MSC-coated cochlear implant. The photograph is taken from the tip of the array. The red layer depicts the boundary between CI surface and alginate coating. The first electrode contact is marked. BDNF-producing MSCs are visible all around the electrode array. The electrode was coated by dip-coating with four inner layers of alginate containing MSCs and three outer layers of cell-free alginate.

The cochlear implants were kindly provided by MED-EL Corp., Innsbruck, Austria. They consisted of a connector, a reference and an active electrode. The active electrode array had two electrode contacts and a marker point to guide insertion at a depth of 3 mm from the tip (Figure 2). The electrode arrays were precoated with poly-L-Lysine (pLL, Sigma-Aldrich, Taufkirchen, Germany), after which they were dipped into 300 μl alginate-MSC solution containing about 500,000 MSCs before subsequently being transferred into a 20 mM BaCl_2_ solution (with 115 mM NaCl and 5 mM L-histidine) to achieve crosslinking of the UHV-alginate-MSC layer, and finally washed with saline solution (0.9% w/v, B. Braun, Melsungen, Germany). In total, four alginate-MSC layers were applied, followed by three outer layers with cell-free alginate to protect the MSCs from the host immune system and to avoid migration of cells (Figure 3).

### Cochlear Surgery

Cochlear implantation was performed in all experimental groups except the NH group. Either a CI (coated or uncoated) was immediately inserted, or one was inserted following insertion of a catheter for purposes of alginate-MSC injection.

The middle ear cavity was opened using a postauricular approach, the cochlea visualized and the round window membrane incised. The CI electrode array was inserted into the scala tympani until the marker point reached the round window niche. Where alginate-MSC was injected (groups: *deaf-alginate-I* and *NH-alginate-I*), a catheter was inserted 3 mm into the scala tympani and the alginate-MSC matrix was injected until the surgeon observed flushing of the medium (pink color) that exited the cochlear while the bony cochlea capsule was being rinsed. The catheter was withdrawn while injection continued, and subsequently the CI was inserted. After CI insertion, the round window niche was filled with TABOTAMP^®^ (Ethicon SARL, Neuchatel, Switzerland) and about two drops of BaCl_2_ were placed on the material using a syringe to induce gelation of the cell-containing UHV-alginate. After 30 min, the TABOTAMP^®^/BaCl_2_ layer was removed. The CI was secured in place and the bulla fenestration site closed using Tetric EvoFlow^®^ (ivoclar vivadent, Schaan, Liechtenstein) in all implantation groups. The reference electrode was placed extratympanically on the bony wall of the bulla and the wound was sutured in two layers.

### Impedance Measurement

Electrode impedances were measured in all implanted animals using a standard MED-EL PULSARci100 stimulator with a HD-CIS 750 pps coding strategy to generate biphasic monopolar pulse trains with a charge of 16 nC, as previously described (Wilk et al., 2016). Starting with the first (apical) contact, impedance was measured three times, followed by three subsequent impedance measurements at the second contact (basal). For data analysis purposes, the mean of the three measurements was taken for each contact (Wilk et al., 2016). *In vivo* measurement of electrode impedance was performed directly after CI insertion and 7, 14, 21, and 28 days postsurgically.

In addition to *in vivo* measurements, the impedance of *n* = 3 electrode arrays was measured *ex vivo* to investigate whether the coating has an impact on electrode impedance. The first measurement without alginate coating was performed directly prior to coating with pLL in phosphate-buffered saline (PBS), because pLL is diluted 1:10 in PBS. The second impedance measurement (without coating) was performed in the MSC medium, and the final measurement (following coating with alginate-MSCs) was carried out in the MSC medium.

### Preparation of Specimen for Histological Analysis

**FIGURE 4:**
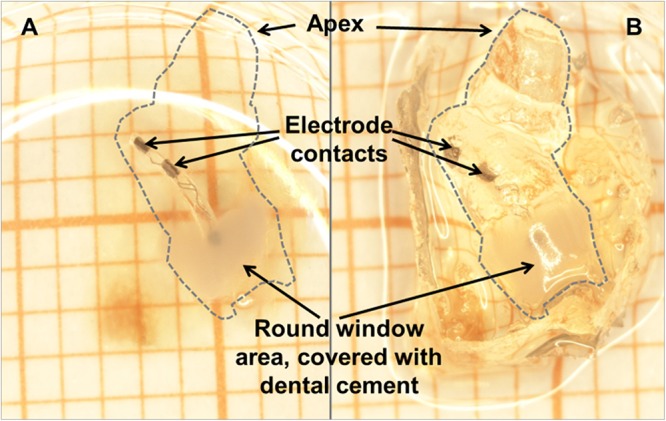
The same cleared cochlea (dashed line) with CI *in situ* in methyl salicylate, benzyl benzoate (MSBB) **(A)** and without MSBB **(B)**, illustrating the total transparency of decalcified cochleae positioned in MSBB **(A)** suitable for confocal laser scanning microscopy of SGNs. The electrode is secured in place at the round window niche with dental cement.

After the final AABR and impedance measurement, the animals received a second injection of the initial anesthesia and were euthanized by transcardial perfusion. Temporal bones were removed (Hütten et al., 2014) and the implant was secured in place at the round window niche using Tetric EvoFlow^®^ (ivoclar vivadent). The duration of fixation was prolonged overnight followed by decalcification for about 3 weeks in 10% ethylenediamine tetraacetic acid-disodium salt (EDTA, Sigma-Aldrich Chemie GmbH, Steinheim, Germany). After dehydration with ethanol, the cochleae were cleared in Spalteholz solution [methyl salicylate, benzyl benzoate (MSBB); Figure 4], placed in self-made glass chambers (Wrzeszcz et al., 2013) and microscopically analyzed.

### SGN Density

Using a Leica TSC SP8 confocal laser scanning microscope and the tissue’s PFA-induced autofluorescence, images were generated at a speed of 400 Hz and 2048 pixels × 2048 pixels with a fivefold object lens or 1024 pixels × 1024 pixels with a 10-fold object lens. Following the previously published protocol, the cochlea was scanned and the images were exported and further processed using ImageJ software (Wrzeszcz et al., 2013). The area of Rosenthal’s canal was traced and the SGNs were automatically counted in the traced area using the Image-based Tool for Counting Nuclei (ITCN) plug-in (Center for Bio-Image Informatics^1^). SGN count was performed on five subsequent images of cochlear cross-sections. The number of SGNs divided by the measured cross-sectional area of Rosenthal’s canal gives the SGN density (cells/10,000 μm^2^). The mean SGN density of the total cochlear length, including all cross-sections of Rosenthal’s canal, was analyzed. The mean SGN density of the (lower and upper) basal turn was also analyzed, but separately.

### Fibrosis

Fibrosis was visually evaluated for one representative image per area to be analyzed. Since no fibrosis was detectable apically from the electrode tip, two areas of the scala tympani were analyzed where the electrode was located. One was the basal part of the cochlea including the area near the round window, and the other was the area of the scala tympani where the electrode tip was located. A subjective evaluation was performed using a ranking system. Scores for subjective ranking were assigned as follows: score 0: no connective tissue; score 1: thin film of fibrosis directly on the electrode surface; score 2: thin fibrous cloudy structures around the electrode; score 3: more prominent cloudy structures around the electrode; score 4: almost the entire investigated area of the scala tympani is filled with fibrous tissue.

### Alginate and Cell Analysis

In one middle ear of the deaf-alginate-MSC injected group, crosslinked alginate was found in the middle ear cavity on experimental day 28 when the specimen preparation was performed. This alginate was transferred into the cell medium and microscopically (CKX53 + Camera XM10, Olympus) analyzed for detection of fluorescent marker protein producing MSCs. Cells with fluorophore expression were deemed to be surviving cells.

### Statistical Analysis

The data were statistically analyzed using the GraphPad Prism^®^5 program.

The relevant data sets (AABR threshold and threshold shift, SGN density, impedances and connective tissue score) were tested for normal distribution of the values, the D’Agostino and Pearson omnibus normality test being used for this purpose. Click-evoked AABR threshold and threshold shift, electrode impedances and SGN densities exhibited normal distribution.

To compare click-evoked AABR threshold shifts between groups and impedances between groups, an unpaired *t*-test was performed. For the purpose of analyzing frequency-specific AABR thresholds on day 0 and day 28, as well as impedance over time, paired *t*-tests were performed.

Spiral ganglion neurons density was then analyzed by applying Bartlett’s Test for Equality of Variances to the sample sets. With a *p*-value of 0.9833, the variances of the SGN densities of all groups were homogeneous. An ANOVA was performed and subsequently the Bonferroni multiple comparison test was used to analyze the variance of independent samples.

The scores yielded by the connective tissue analysis were not distributed normally. The Kruskal–Wallis test was performed to compare these scores between groups, and the Wilcoxon matched-pairs test was used to compare basal and apical fibrosis within one experimental group.

The significance levels determined were defined as follows:

• *p* > 0.05 = not significantly different (ns).
• *p* < 0.05 = significantly different (^∗^).
• *p* < 0.01 = highly significantly different (^∗∗^).
• *p* < 0.001 = most significantly different (^∗∗∗^).

In the following sections, the data are represented as mean ± standard error of mean (SEM) for each experimental group.

## Results

### AABR

A reference AABR using click stimuli was performed in all animals prior to their inclusion into the study to confirm that physiological hearing function was present. All animals’ hearing threshold (based on click-evoked potentials) was 80 dB att. (=40 dB SPL) or lower, and therefore all animals showed normal hearing as defined by previous studies.

An additional AABR measurement was performed on experimental day 0 in animals 1 week after treatment with kanamycine and furosemide, the aim being to verify the success of the deafening method. All animals receiving ototoxic drugs were deaf and therefore underwent cochlear implantation.

To investigate whether alginate injection may have an impact on residual hearing in implanted subjects, click- and frequency-specific hearing thresholds were analyzed for normal-hearing animals unilaterally provided with a cochlear implant (*NH-CI*), and contralaterally injected with alginate-embedded mesenchymal stem cells (MSCs) followed by CI insertion (*NH-alginate-I*).

**FIGURE 5:**
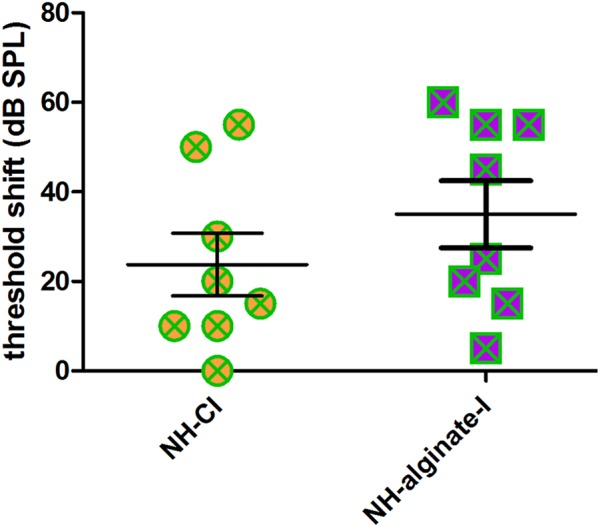
The mean hearing threshold shift (click-evoked) of normal-hearing ears implanted with an uncoated cochlear implant (NH-CI) or of normal-hearing ears receiving an alginate-MSC injection followed by insertion of an uncoated cochlear implant (NH-alginate-I) did not differ 28 days after implantation. Each data point represents the threshold shift of click-evoked AABR in one ear.

Using click-evoked AABR, a mean threshold shift (difference between day 0 threshold before surgery and day 28 threshold before perfusion) of 23.75 ± 19.78 dB SPL was detected in *NH-CI* ears. The same animals received a contralateral injection of UHV-alginate-MSCs; a CI was subsequently inserted and the UHV-alginate was crosslinked for 30 min using BaCl_2_. These ears had a threshold shift of 35.00 ± 21.21 dB SPL (*NH-alginate-I*). No statistically significant differences in mean hearing loss between both experimental groups were observed (Figure 5).

**FIGURE 6:**
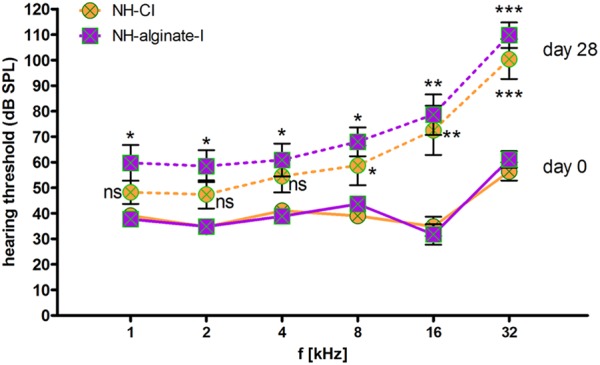
Frequency-specific hearing thresholds in normal-hearing ears. The data shown are means ± SEM of data for all ears (*n* = 8) within the various experimental groups. Continuous lines: day 0 thresholds before implantation. Dashed lines: Thresholds 28 days after implantation. Orange: Normal hearing with CI (NH-CI); purple: Normal hearing with alginate-MSC injection and subsequent CI insertion (NH-alginate-I). Significant differences between initial hearing thresholds and the thresholds determined after 28 days of implantation are depicted above (NH-alginate-I) or below (NH-CI) the experimental condition in question. In both experimental groups, the hearing threshold at the higher frequencies (8, 16, and 32 kHz) increased significantly after implantation. At 4–1 kHz, cochlear implantation did not affect the threshold significantly, but alginate injection with subsequent CI insertion resulted in a significantly increased threshold at all frequencies. ns = not significat; ^∗^*p* < 0.05; ^∗∗^*p* < 0.01; ^∗∗∗^*p* < 0.001.

Analysis of frequency-specific thresholds revealed a significant increase in high-frequency thresholds (8, 16, and 32 kHz) in both experimental conditions, namely both CI insertion and alginate injection followed by cochlear implantation (Figure 6). At 32 kHz, the mean threshold shift after 28 days of implantation was 44 ± 17 dB (NH-CI) and 48 ± 13 dB (NH-alginate-I). With increasing distance from the round window, the threshold shift decreased in both conditions, from 37 ± 26 dB (NH-CI) and 47 ± 25 dB (NH-alginate-I) at 16 kHz to 20 ± 23 dB (NH-CI) and 24 ± 21 dB (NH-alginate-I) at 8 kHz, and 13 ± 19 dB (NH-CI) and 22 ± 22 dB (NH-alginate-I) at 4 kHz; in NH-CI treated ears, no significant difference in hearing thresholds (compared with the initial condition) was observed. At the lowest frequencies (i.e., 1 and 2 kHz), the hearing threshold on day 0 and day 28 in UHV-alginate- injected and cochlear-implanted ears (NH-alginate-I) – but not in only cochlear-implanted ears (NH-CI) – differed significantly (NH-CI mean threshold at 1 kHz: d0 39 dB and d28 48 dB; at 2 kHz: d0 35 dB and d28 47 dB; NH-alginate-I mean threshold at 1 kHz: d0 38 dB and d28 60 dB; at 2 kHz: d0 35 dB and d28 58 dB).

### SGN Survival

**FIGURE 7:**
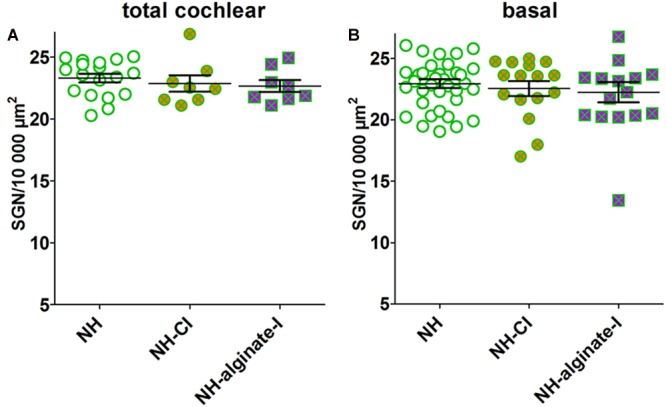
Mean spiral ganglion neuron density in normal-hearing ears without further intervention (NH; *n* = 9 animals, i.e., *n* = 18 ears), with cochlear implantation (NH-CI; *n* = 8 ears) and with injection of alginate-MSCs followed by CI insertion (NH-alginate-I; *n* = 8 ears) did not differ over the entire length of the cochlea **(A)** and for the basal region (lower and upper basal turn) **(B)**. Each data point in **(A)** represents the mean SGN density of one animal over the full length of the cochlea. Each data point in B represents the mean SGN density of the lower basal or upper basal cochlear turn of one experimental animal. ns = not significat; ^∗∗^*p* < 0.01; ^∗∗∗^*p* < 0.001.

In normal-hearing guinea pigs (NH, *n* = 9 animals, *n* = 18 ears), a mean neuronal density of 23.31 ± 0.34 spiral ganglion neurons (SGN)/10,000 μm^2^ was detected. Implantation of a CI or injection of UHV-alginate-MSCs followed by CI insertion in normal-hearing/non-deafened animals did not affect the SGN density compared with NH when the total cochlea is analyzed (NH-CI: 22.67 ± 0.48 SGN/10,000 μm^2^, *n* = 8; NH-alginate-I: 22.87 ± 0.65 SGN/10,000 μm^2^; *n* = 8; Figure 7A) or when the lower and upper basal region of the cochlea is investigated (Figure 7B) (ANOVA, total cochlea: *p* = 0.57; basal: *p* = 0.64).

**FIGURE 8:**
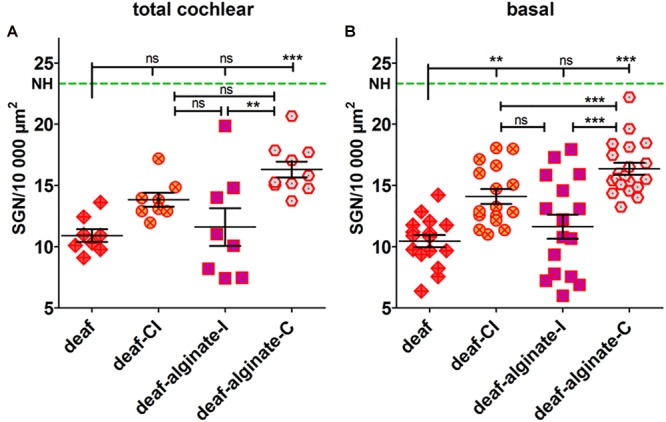
The SGN density (number of surviving SGNs in 10,000 μm^2^) was influenced by the different interventions tested when data were collected for the total length of the cochlea **(A)** and, even more markedly, when the basal cochlear region was analyzed separately **(B)**. The deafening procedure resulted in a significantly reduced SGN density compared with the normal-hearing control (NH, green dashed line). Cochlear implantation preserved SGNs from degeneration in the basal region. Coating the CI with alginate-MSCs (deaf-alginate-C) significantly preserved the SGNs from degeneration in deafened animals; the injection (deaf-alginate-I), however, did not. Each data point in **(A)** represents the mean SGN density of one animal over the full length of the cochlea. Each data point in **(B)** is the mean SGN density of the lower basal or upper basal cochlear turn of one experimental animal. ns = not significat; ^∗∗^p < 0.01; ^∗∗∗^p < 0.001.

The analysis of variance of the mean SGN densities in deafened ears showed highly significant differences both for the total cochlear (*p* = 0.0005) and for the basal region (*p* < 0.0001) when compared with NH. Applying the Bonferroni multiple comparison test, the mean SGN densities of the deafened groups were tested for difference. The deafening procedure resulted in a significantly reduced mean SGN density of 10.91 ± 0.52 SGN/10,000 μm^2^ over the entire length of the cochlea (Figure 8A). With 13.84 ± 0.56 surviving SGN/10,000 μm^2^, CI insertion evidently did not change neuronal survival compared with the non-implanted deafened ears, if measurements over the entire length of the cochlea are included. Four weeks after implantation of alginate-MSC-coated CIs into deafened ears, the SGNs were significantly protected compared with deafened controls (deaf-alginate-C: 16.30 ± 0.64 SGN/10,000 μm^2^ vs. deaf: 10.91 ± 0.52 SGN/10,000 μm^2^, *p* < 0.05). The injection of MSC-containing alginate (deaf-alginate-I: 11.61 ± 1.54 SGN/10,000 μm^2^) did not affect SGN survival compared with the deafened, or deafened and CI-implanted, ears, but resulted in significantly lower SGN survival than where ears were implanted with an alginate-MSC-coated CI.

Focusing on the basal cochlear region (lower basal and upper basal cross-section of Rosenthal’s canal) where injection and implantation takes place, even more prominent differences are evident between the treatment strategies (Figure 8B). Cochlear implantation (*per se*) resulted in better SGN preservation than no intervention at all (deaf-CI: 14.10 ± 0.60 SGN/10,000 μm^2^ vs. 10.46 ± 0.50 SGN/10,000 μm^2^, *p* < 0.01). Protection of SGNs from degeneration by coating the CI with MSC-containing alginate resulted, in the basal cochlear region, in a SGN density of 16.36 ± 0.048 SGN/10,000 μm^2^, and was significantly improved compared with all other conditions (*p* < 0.001). No difference was observed between cochlear-implanted ears and those receiving an alginate injection before cochlear implantation (deaf-CI: 14.10 ± 0.60 SGN/10,000 μm^2^ vs. deaf-alginate-I: 11.64 ± 0.98 SGN/10,000 μm^2^; ns).

### Impedance

**FIGURE 9:**
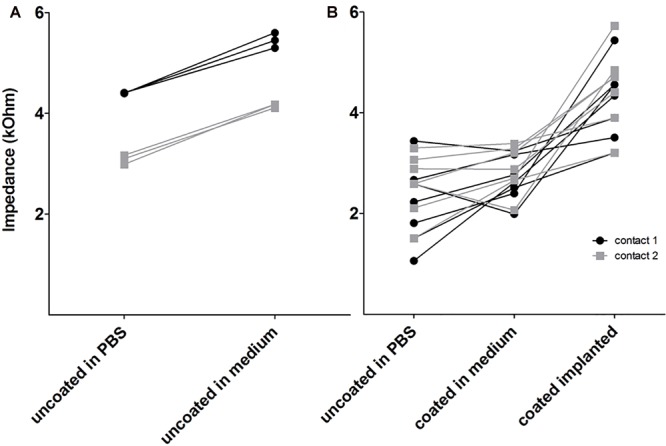
Electrode impedance on electrode contact 1 (apical) and 2 (basal) in PBS and MSC medium before coating **(A)** and after coating **(B)** in medium and *in vivo* (coated implanted). Electrode impedance of uncoated contacts was increased in MSC medium compared with PBS **(A)**. The coating did not affect impedance but after implantation, all impedances were increased **(B)**.

To investigate whether coating with alginate-MSCs had an impact on electrode impedance, coated electrodes had to be measured in the MSC medium to avoid damage to these cells and alginate destruction. Comparative measurements were made of the electrode impedance of arrays placed in the MSC medium and those placed in PBS: for both electrode contacts, impedance was found to be lower in PBS than in the MSC medium (Figure 9A; those data are not included in Figure 9B). Electrode impedances of contacts in the MSC medium were not affected by alginate-MSC coating as compared with impedance of the same contact before coating (Figure 9B; mean of contact 1 and 2 for uncoated in PBS: 2.38 ± 0.19 kΩ versus alginate-MSCs coated in medium: 2.77 ± 0.11 kΩ). In contrast to impedances measured *ex vivo* in the MSC medium, impedances measured *in vivo* directly after surgery were significantly increased (4.36 ± 0.20 kΩ, *p* < 0.001).

Change over time in impedance *in vivo* did not differ between experimental groups. This is mainly due to the high variability in each group (Supplementary Figure S1).

**FIGURE 10:**
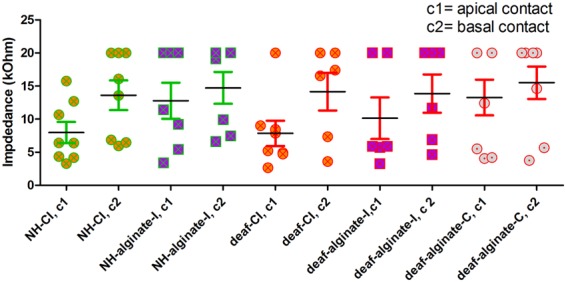
Electrode impedance on experimental day 28 did not differ between experimental groups.

Comparison of final electrode impedances on experimental day 28 revealed a statistically not significant tendency toward increased impedance at contact 2 (basal) in comparison with contact 1 (tip) in all groups. No differences in electrode impedance between groups were found (Figure 10).

### Fibrosis

**FIGURE 11:**
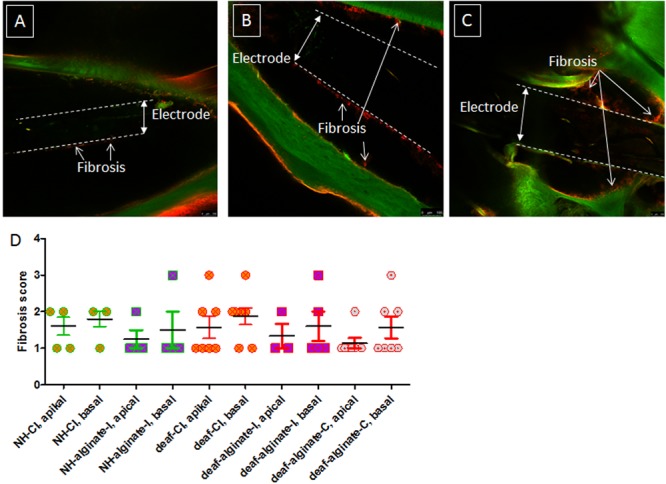
Fibrosis score of implanted ears for all experimental groups. Representative images of scores 1 **(A)**, 2 **(B)**, and 3 **(C)** are given. No images of score 0 and score 4 are shown, since all cochleae were affected to some extent by fibrosis and none of them was densely packed with fibrotic tissue. The extent of fibrosis did not differ between groups **(D)**.

Fibrosis around the electrode array was visible in all cochleae analyzed. No fibrosis was detectable apically from the electrode tip. None of the ears showed an absence of fibrosis (score 0), and none was affected by massive fibrosis with a score of 4. Figure 11 includes representative images for scores 1, 2, and 3. No difference between alginate-injected cochleae or cochleae with insertion of alginate-coated CI or uncoated CI was observed (Figure 11).

### Alginate and Cell Analysis

**FIGURE 12:**
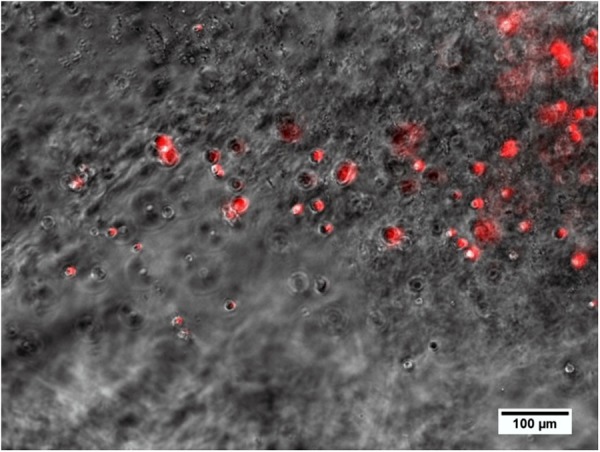
Alginate including spherical MSCs explanted from the middle ear 28 days after injection and crosslinking in an animal. Several MSCs still produce the red fluorescence marker protein tdTomato, which is associated with the genetic modification for BDNF-overexpression and is an indicator for living cells.

The explanted alginate from one animal after 28 days of injection included living MSCs (Figure 12).

## Discussion

To determine the neuroprotective effect of brain-derived neurotrophic factor endogenously overexpressed from infected mesenchymal stem cells (MSCs), two different application methods were evaluated in systemically deafened guinea pigs. The BDNF-overexpressing MSCs were encapsulated in a UHV-alginate matrix and were either injected into the scala tympani or used to coat the cochlear implant array.

### Neuroprotection

Cochlear implantation resulted in significantly increased spiral ganglion neuron survival in the basal region compared with deafened ears that were not further treated (Figure 8). A greater decrease in SGN density was to be expected due to the fact that the cochlear-implanted ears were locally manipulated, in contrast to the deafened ears which were not opened at all. It is possible, however, that merely the activation of the electrode for the purpose of the weekly impedance measurement itself resulted in a neuroprotective effect. It is known that electrical stimulation may have a protective effect on auditory neurons (Scheper et al., 2009; Leake et al., 2013; Shepherd et al., 2018). The electric fields increase gene expression for, and synthesis of, growth factors (Aaron et al., 2004), which may lead to neuroprotection through autocrine and paracrine neurotrophic signaling (Hansen et al., 2001). To date, exact parameters for electrical stimulation of the SGNs that result in reliable neuroprotection have not been defined. There are indications that even only short-term electrical stimulation may lead to increased SGN survival. This application of ‘short-term’ stimulation was for 2.3 h during weekly electrical ABR measurements in guinea pigs (Mitchell et al., 1997), and for 8–88 h over a period of 8.5–9.6 months in cats (Konerding et al., 2017). It may be that, by contrast, neuronal protection in the present study was initiated by electrical stimulation during impedance measurement, which is very brief and involves only a matter of seconds. This is a very interesting finding which should be investigated further in future projects.

Coating the CI with alginate containing MSCs that continuously secrete BDNF additionally increased SGN survival in deafened animals compared to cochlear implantation without alginate-MSC functionalization (Figure 8). This effect is significant for the basal region (*p* < 0.001). Compared with deafened-only ears, this effect is evident in terms of the mean SGN density of the entire cochlea, and also if the focus is on the basal region only. This coating would therefore seem to be a feasible method of applying BDNF-overexpressing MSCs into the inner ear for chronic growth factor therapy. It is known from previous *in vitro* experiments that 50 ng/ml exogenous, recombinant human BDNF is optimal in order to preserve murine SGNs from degeneration, and that lower concentrations result in lower numbers of surviving neurons (Wefstaedt et al., 2005). To date, *in vivo* BDNF delivery has involved an osmotic pump or carrier matrices that deliver it into the inner ear or onto the round window. Ramekers et al. (2015) used pumps with a flow rate of 0.25 μl/h filled with 100 μg/ml BDNF, resulting in a calculated total quantity of BDNF infused into guinea pig cochleae after 28 days of about 17 μg. The same concentration of BDNF, i.e., 100 μg/ml, delivered into the inner ear using a pump was examined as to its biological effect by Miller (in combination with FGF) and by Miller et al. (2007) and Agterberg et al. (2009). Other studies have used much lower concentrations in guinea pigs [50 ng/ml pump-based delivery (Miller et al., 1997), or gelfoam cubes (1 mm^3)^ infiltrated with 6 μg BDNF/ml saline placed on the round window (Havenith et al., 2010)] or rat [5.4 μg/ml pump-based delivery (McGuinness and Shepherd, 2005)] which also effectively preserved SGNs from degeneration. We know that the BDNF produced by the encapsulated hMSCs and released from the UHV-alginate was in the pg/ml-range and protected SGNs from degeneration *in vitro* (Schwieger et al., 2018). Although we did not measure the amount of BDNF released from the alginate coating *in vivo*, we speculate that the BDNF concentration in the inner ear was also in the pg/ml-range. If this is indeed the case, then our study reports a neuroprotective effect using a very low dose of BDNF compared with experiments described in the literature. It must be noted, however, that all these previous studies reported the BDNF concentration in the primed pumps or matrixes but did not measure the final concentration in the perilymph. There are initial reports on the pharmacokinetics of glucocorticoids (Salt and Plontke, 2018), but relevant studies regarding growth factor uptake, distribution and stability in the inner ear are still pending. It remains virtually impossible to state which BDNF concentration is required *in vivo* to achieve significant protection of SGN, since relevant pharmacokinetic studies are lacking. It should also be mentioned that there are indications that endogenous growth factors, as used in the present study, may be more potent for neuronal protection than exogenous factors, due to increased bioactivity of the endogenously produced growth factor. For erythropoietin (EPO), a profound structural difference between human endogenous and various pharmaceutical preparations of human recombinant erythropoietins have been shown (Reichel, 2011), which may lead to different biological activity. This effect may also apply for endogenous and recombinant BDNF. Before translating any approach for growth factor delivery into clinical practice, well-planned and well-performed studies on their pharmacokinetics are a prerequisite.

In contrast to coating the CI with alginate-MSCs, injection of the alginate-MSC matrix with subsequent CI insertion did not affect SGN density compared to deafened-only ears (Figure 8). Since increased SGN survival was observed in the basal region of ears implanted with a CI, it would appear that alginate injection diminishes the effect of cochlear implantation. The reduced SGN survival observed in the injected alginate-MSC cohort (compared to the cohort with alginate-MSC coating on the electrode array) is caused by the injection technique. Although histological analysis did not include seeking for trauma of the inner ear structures, we know from the electrophysiological data that there is a functional impact on the inner ear that is mediated by injection of alginate-MSCs. Since we did not observe a difference in fibrous tissue growth between the groups, we speculate that the reason for the absence of a neuroprotective effect is not increased tissue trauma but physical impact on the delicate inner ear structures.

### Residual Hearing

To investigate whether the hearing threshold may be influenced by alginate-MSC injection, we provided one experimental group with unilateral CI and alginate-MSC injection combined with subsequent CI insertion in the contralateral ear. Cochlear implantation resulted in a shift of 23 ± 20 dB SPL in click-evoked AABR threshold levels. In comparison to CI insertion with previous alginate injection, where the threshold increased by 35 ± 21 dB, no significant differences were detectable (Figure 5). However, the recording of click stimuli gives the lowest threshold detectable over the entire frequency range of the cochlea. To investigate whether there are differences between the high- (CI and alginate injection) and the low-frequency regions of the ear (no manipulation, region of physiologically functioning cochlear in patients with residual hearing), frequency-specific thresholds were measured. In both implantation modes – with and without additional alginate injection – high-frequency (32, 16, and 8 kHz) hearing ability was significantly reduced compared with the initial thresholds measured before cochlear manipulation. At 4–1 kHz, cochlear implantation did not modify the threshold significantly, but alginate injection with subsequent CI insertion resulted – across all frequencies – in a significantly increased threshold (Figure 6). Hearing preservation after cochlear implantation is affected by factors including insertion depth, length and mechanical characteristics of the electrode in use, surgical technique used (Lenarz and Scheper, 2015), insertion angle (Helbig et al., 2018) as well as insertion trauma, foreign-body reaction, electrode-neuron interfacing, and long-term stability of electrode position and function (Lenarz and Scheper, 2015). The high-frequency threshold increase observed in this study may be due to the presence of the CI inside the scala tympani, the insertion angle or depth, as well as to fibrous tissue growth around the electrode. It can, however, be stated that this high-frequency hearing loss is not clinically relevant, since the most important prerequisite for cochlear implantation is high-frequency hearing loss. Furthermore, we observed a significant low frequency threshold shift in all animals receiving an alginate injection. This loss of hearing in a cochlear region where no manipulation was performed may be due to the filling of the scala tympani with viscous alginate. This may lead to interference with the pressure wave in the perilymph or with basilar membrane movement, which may in turn lead to decreased activation of the high-frequency areas of the inner ear.

We did not investigate a potential effect of alginate-MSC-coated CIs on the hearing threshold. It cannot be ruled out that such coating affects the hearing ability by inducing additional swelling, for example in general, alginate hydrogels are osmotically active and swell in, for example, low-pH environments or in hypotonic solutions (Bajpai and Sharma, 2004). The alginate hydrogel used in this study was produced using isoosmolaric reagents (storage solution and cross-linked solution). In a previous study, Ehrhart et al. (2013) demonstrated the stable volume (low swelling behavior) of such UHV-alginate hydrogels in isoosmolaric media over time. However, for reasons of patient safety, the swelling behavior in perilymph has to be investigated in detail in future studies.

### Alginate and Cell Analysis *in situ*

The injection of alginate-MSCs was visually monitored and discontinued as soon as the surgeon observed the phenol-red-colored alginate (due to the matrix formed with the MSC medium) exiting the cochlea. There was, however, one individual in which UHV-alginate was found in the middle ear cavity. By means of microscopic analysis, it was proven that the MSCs survived (as indicated by red fluorescence) in alginate for 28 days *in vivo* (Figure 12).

No information about the alginate-MSCs located in the scala tympani is generated by the present study, due to the fact that dehydration is a prerequisite for preparation of specimens for histological analysis of SGNs. This is not only the case for the method used in this study, but also for established procedures such as paraffin or plastic embedding (Scheper et al., 2017). Dehydration erases the alginate, so that the MSCs no longer remain *in situ* and cannot be visualized in the scala tympani. If electrodes had been explanted to evaluate the coating, it would not have been possible to compare fibrosis between CI-explanted ears and other ears where the electrode remained *in situ*; this is because it cannot be ruled out that, together with the electrode, fibrous tissue is additionally translocated or even extracted from the scala. Potential future studies could involve implanting additional animals, focusing only on investigation of alginate coating stability. Alternatively, the electrode array could be left *in situ*, and parts of the bony cochlear wall removed in order to investigate the electrode and its coating *in situ*, with subsequent dehydration for histological processing.

### Fibrosis

The amount of fibrosis around the implanted arrays did not differ between the experimental groups, suggesting that the alginate – whether in the form of coating or an injection – does not increase the activation of the host immune system. It has previously been shown that the UHV-alginate is biocompatible (Schneider et al., 2005; Zimmermann et al., 2007), and here we show for the first time that this is also the case for application in the inner ear.

### Electrode Impedance – Effect of Coating

Electrode impedance of uncoated contacts was increased in the MSC medium compared with PBS. These measurements were started in PBS, with three consecutive measurements performed; only afterwards were impedance levels measured in the MSC medium, again three times. Electrode impedance decreases after activation in an animal model (Wilk et al., 2016) and in humans (Hu et al., 2017). Therefore, the impedances were expected to be lower in the MSC medium than in PBS. Since the medium is protein-rich, these proteins may have attached to the contact surface and increased impedance levels. Since the coated electrodes in the MSC medium had the same impedance levels as uncoated electrodes in PBS, it can be speculated that, with this coating, protein attachment was prevented, so that it positively influenced electrode impedance. After implantation, all impedance levels of the coated electrodes were significantly higher; this finding was expected, since it is well known that electrode impedance rises after implantation (Paasche et al., 2006) compared with impedance measured *ex vivo*.

### Electrode Impedance – Effect of Experimental Condition Over Time

Electrode impedance levels were measured weekly in all implanted ears over the experimental period of 28 days (Supplementary Figure S1). Changes in impedance over time did not differ between groups, suggesting that where the coating or scala tympani fills with the alginate-MSC matrix, this may not have a negative effect on electrode impedance.

## Conclusion

Coating of the electrode array with BDNF-producing mesenchymal stem cells embedded in UHV-alginate has the effect of protecting spiral ganglion neurons from degeneration in systemically deafened animals. Such coating is superior to alginate-MSC injection, which did not affect the SGNs and which resulted in increased hearing loss compared with cochlear implantation alone in normal-hearing animals.

Further research and development are needed before this concept can be tested in clinical trials. Additional studies are needed into how long MSCs survive *in vivo* in the alginate coating, and into whether the neuroprotective effect can be sustained for longer periods. Additionally, chronic electrical stimulation should simultaneously be applied: to mimic the situation in CI patients, and to investigate the influence of electrical stimulation on MSCs, coating stability and the combined effect of electrical stimulation and MSC-produced BDNF on SGNs.

## Ethics Statement

All animal procedures were performed in accordance with the European Council directive (2010/63/EU). The protocol was approved by the Local Institutional Animal Care and Research Advisory Committee (IACUC) and permitted by the local authority [Lower Saxony State Office for Consumer Protection, Food Safety, and Animal Welfare Service (LAVES); approval number 17/2396].

## Author Contributions

VS, TL, and AHo conceived and designed the experiments. MG and AS produced the UHV-alginate used in the study. AHo and AHa isolation, expansion, transduction, and characterization of MSCs. VS, JS, and PH performed *in vivo* experiments. VS, JS, and CP participated in the processing of the cochleae for histology. CP blinded to the different groups and performed the CLSM and analyzed these data. PH designed and implemented software for AABR analysis. VS and PH analyzed the AABR data. JS impedance data. VS and JS wrote the first version of the manuscript. All authors participated in the reviewing and rewriting of the manuscript. All authors read and approved the final manuscript.

## Conflict of Interest Statement

The authors declare that the research was conducted in the absence of any commercial or financial relationships that could be construed as a potential conflict of interest.

## Footnotes

1 https://imagej.nih.gov/ij/plugins/itcn.html
