# Supplementary material for: Stem Cell Based Drug Delivery for Protection of Auditory Neurons in a Guinea Pig Model of Cochlear Implantation
Source: Front Cell Neurosci. 2019 May 14;13:177. doi: 10.3389/fncel.2019.00177 (PMC6527816; doi:10.3389/fncel.2019.00177)
Supplement: Supplementary file 1 [file Table_1.DOCX]

Supplementary Material

# Supplementary Data

## Supplementary Figures

**Supplementary Figure 1.** Mean impedance development of electrode 1(apical) and 2 (basal) over the experimental time period of 28 days for all experimental groups being implanted with a CI. No differences in impedance development were observed between groups.
